# The active pulling technique to solve microcatheter-uncrossable lesions in retrograde chronic total occlusion percutaneous coronary intervention

**DOI:** 10.1007/s10554-024-03068-0

**Published:** 2024-02-26

**Authors:** Hongmin Zhu, Xinyong Cai, Yuliang Zhan, Lang Hong

**Affiliations:** 1 Jiangxi Medical College, Nanchang University, Nanchang, 330046 Jiangxi China; 2 Department of Cardiology, Jiangxi provincial People’s Hospital, The First Affiliated Hospital of Nanchang Medical College, No. 256, Fenghebei Avenue, Honggutan District, Nanchang, 330006 Jiangxi China

**Keywords:** Active pulling technique, Microcatheter-uncrossable lesions, Chronic total occlusion, Retrograde approach

## Abstract

Background: It is not uncommon to encounter retrograde microcatheter-uncrossable lesions in retro-recanalization of Chronic Total Occlusion (CTO) cases, existing solutions were time-consuming or complicated to operate. Therefore, the present study aimed to propose and evaluate the feasibility, safety of a novel technique termed Active Pulling retrograde microcatheter crossing Technique (APT) during retrograde CTO percutaneous coronary intervention (PCI). Methods: We retrospectively collected retrograde CTO-PCI cases from February 2017 to April 2023, only cases with the retrograde wire successfully crossed the CTO lesion were analyzed. The baseline clinical characteristics, angiographic characteristics, procedural details, and in-hospital major adverse cardiac events (MACEs) were compared. Results: A total of 80 CTO cases were divided into the APT group and the non-APT group according to whether the APT was applied in the procedure. The APT group had a higher rate of device success than the non-APT group (100% vs. 85%, *P* = 0.013), with shorter duration (5.3 ± 3.8 vs. 18.6 ± 5.9 min, *P* < 0.001) and a smaller number of retrograde microcatheters were used (*P* < 0.001). In the APT group, the average air kerma radiation exposure was lower (2.7 ± 1.2 vs. 4.3 ± 1.7 Gy, *P* < 0.001), the fluoroscopy time (69.0 ± 15.0 vs. 88.1 ± 18.9 min, *P* < 0.001) and the procedure time (116.2 ± 22.2 vs. 131.6 ± 28.7 min, *P* = 0.009) was shorter than the non-APT group. The technical success rate of both groups reached 100% while the procedure success rate was higher in the APT group than the non-APT group (100% vs. 85%, *P* = 0.13). Conclusions: The APT is an easy and safe technique that can greatly improve procedural efficiency without adding other instruments, and allows the retrograde microcatheter to quickly crossing the CTO body after successful retrograde wire externalization.

## Introduction

The retrograde approach to chronic total occlusion (CTO) percutaneous coronary intervention (PCI) was an effective method to improve the success rate of CTO PCI and had led to its worldwide adoption [1–4]. When performing retrograde CTO PCI, a central goal is for the retrograde wire (RW) to cross the occlusion and, even better, enter the antegrade guide catheter [5]. It is not necessary but preferable to keep the retrograde microcatheter (RM) following the RW across the occlusion for an exchange to an externalization wire [e.g. RG3 (Asahi intecc) or R350 (Vascular Solutions)] or for the rendezvous technique of threading an antegrade wire through the RM and crossing the CTO [6]. However, it happens from time to time that the RM was unable to cross the CTO lesion even though the RW had crossed, yet current algorithms or existing techniques had paid little attention to this issue [7, 8]. When facing such microcatheter-uncrossable dilemma, traditional method (the trapping balloon technique) involves a “push” of the RM relying upon the support of the retrograde guide, perhaps with the distal end of the RW trapped or snared in the antegrade guide to secure a rail. Such method is the easiest but not the most effective way because the “push” from the retrograde side travels a long, tortuous collateral channel to aid in RM crossing the lesion. Changing another new RM might be a good choice, but it is time-consuming and costly. Both Rendezvous in Coronary Technique and Intracoronary Rendezvous technique provide possible ways of advancing the retrograde/antegrade guidewire into an antegrade/retrograde microcatheter inside the CTO lesion, while the Tip-In Technique refers to delivering the RW into the antegrade microcatheter in the curved segment of the antegrade guide catheter after RW successfully entered the guide [9–11]. Still, all these techniques are easy to learn, but hard to master. Therefore, we proposed a simple Active Pulling retrograde microcatheter crossing Technique (APT) to solve microcatheter-uncrossable lesions in retrograde CTO PCI.

## Materials and methods

### Study population

We retrospectively collected CTO-PCI cases in our department from February 2017 to April 2023. All included CTO cases for the present study must have employed a retrograde approach, and only cases with the RW successfully crossed the occluded lesion were included. Those cases who were treated with the APT were assigned to the APT group. All patients were administered dual antiplatelet therapy before PCI. During the procedure, unfractionated heparin was given to all patients with a bolus of 100U/kg, and the activated clotting time was then monitored between 350 and 400s. All the procedures were performed by experienced operators. The measurement of cardiac troponin I (cTnI) before the PCI procedure was recorded and repeated 6–12 h after the procedure. Once the later cTnI value is rising, further sampling would be tested to document the peak cTnI value. All participants provided informed consent, including consent for the procedure, following the Helsinki Declaration as revised in 2013. This study was approved by the Ethics Committee of Jiangxi provincial People’s Hospital Affiliated to Nanchang University, The First Affiliated Hospital of Nanchang Medical College.

### Technique description

**Fig. 1:**
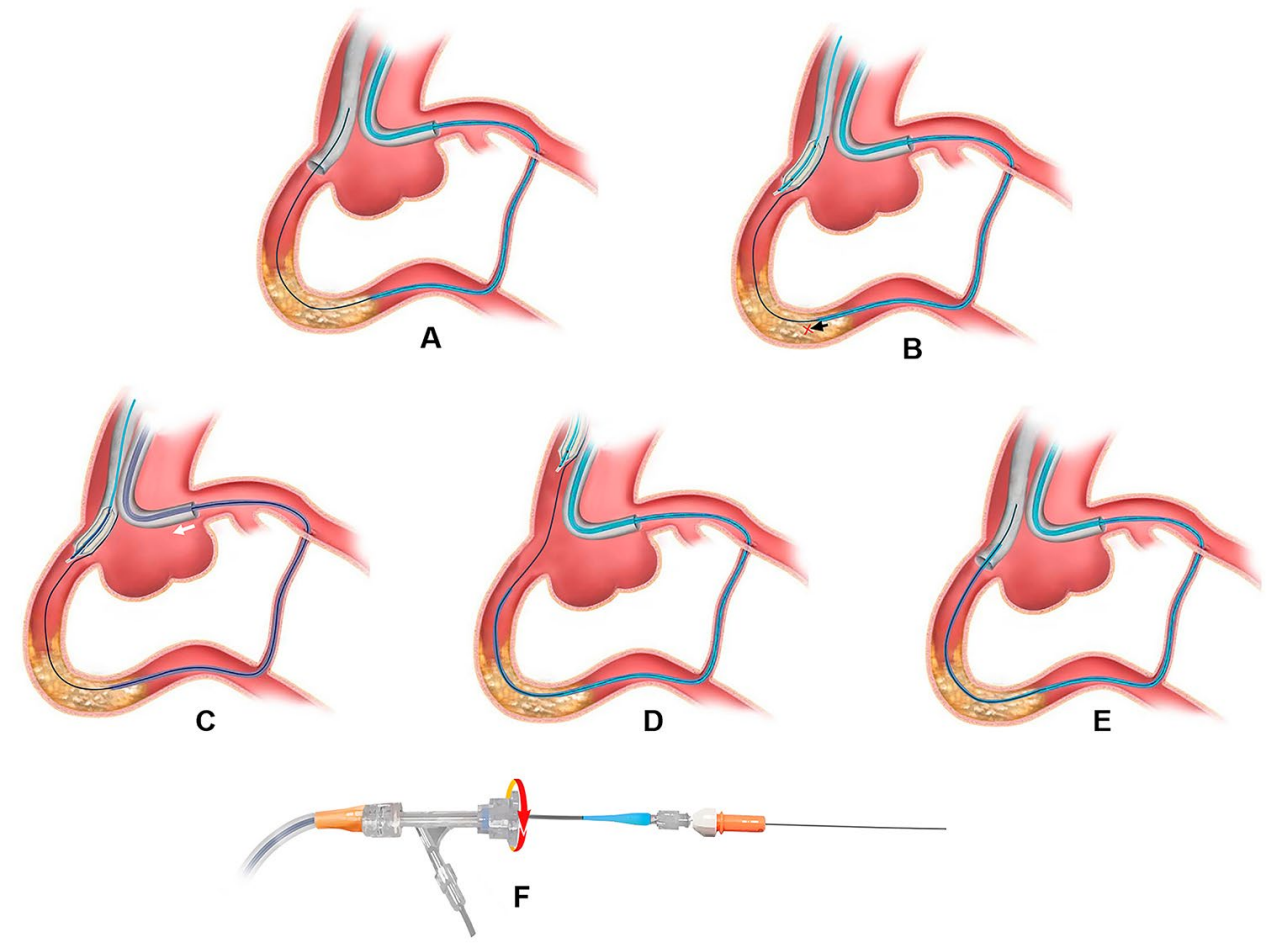
The schematic diagram of the APT. Legends: (**A**) The retrograde wire crossed a RCA CTO lesion and entered into the antegrade guide catheter. (**B**) The retrograde microcatheter failed to cross the lesion (black arrow) after the retrograde wire was anchored in the antegrade guide catheter with an inflated balloon. (**C**) Disengaging the retrograde guide catheter from the coronary ostium (white arrow) to avoid ostial injury. (**D**) Pulling the antegrade guide catheter and the retrograde wire upwards so that the retrograde microcatheter was pulled through the CTO lesion. (**E**) The antegrade guide catheter re-engaged the RCA and the retrograde microcatheter was advanced. (**F**) A torque device was installed at the entry port of the retrograde microcatheter to interlock it and the retrograde wire. APT refers to the active pulling retrograde microcatheter crossing technique

**Fig. 2:**
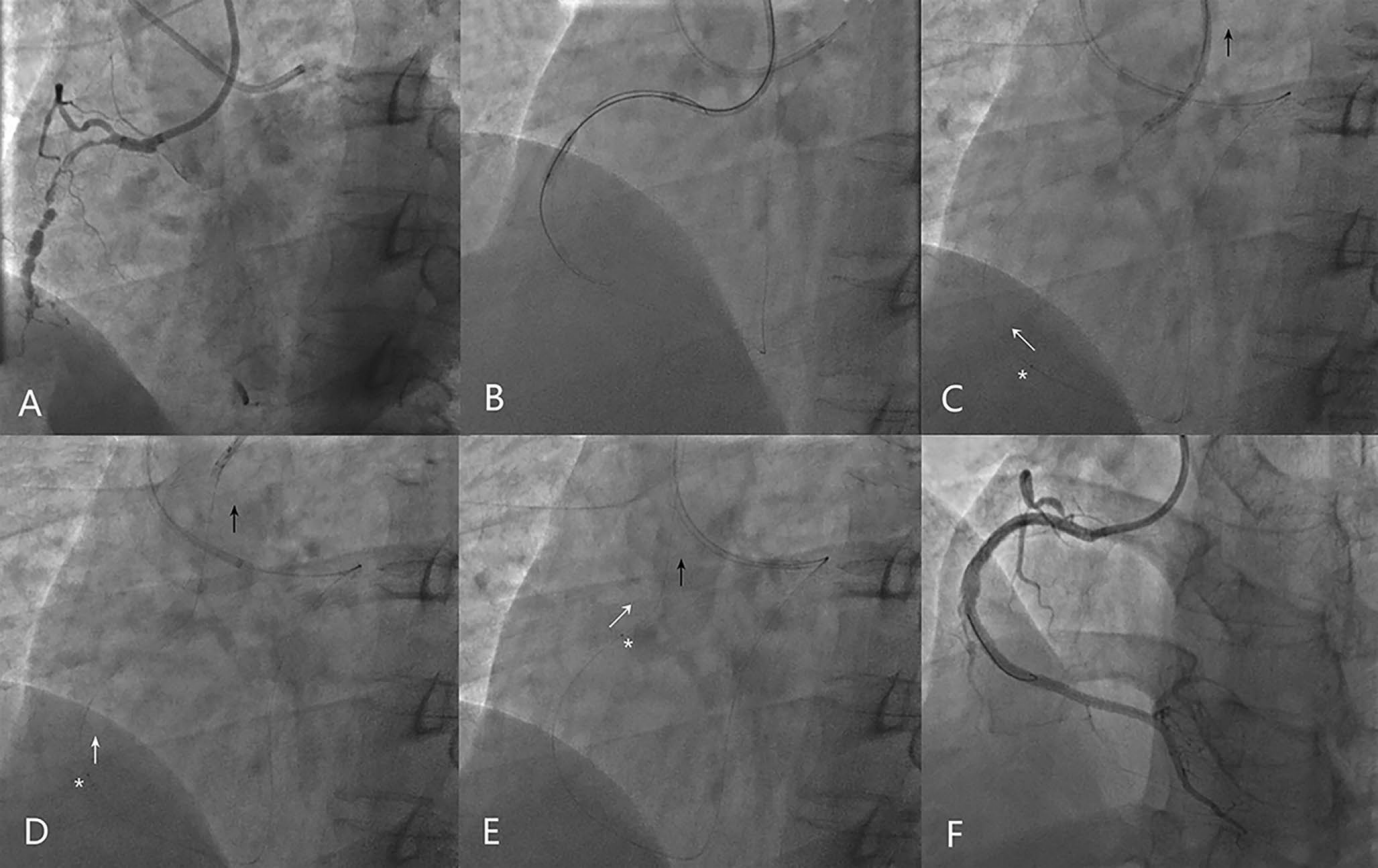
An example of the APT implemented via a septal collateral channel in a retro-recanalization CTO case. (**A**) The bilateral angiography showed a RCA CTO lesion (J-CTO score = 4). (**B**) The RW successfully entered the antegrade guiding catheter. (**C, D**) The RM (white star) failed to cross the CTO body. The antegrade guide catheter and RW was together pulled to help the RM cross the occlusion. (white arrow and black arrow showed the motion of RM and antegrade catheter, respectively). (**E**) The RM (white star) successfully crossed the lesion. (**F**) The final angiogram showed successful recanalization of the CTO lesion. RW refers to the retrograde wire. RM refers to the retrograde microcatheter. APT refers to the active pulling retrograde microcatheter crossing technique

**Fig. 3:**
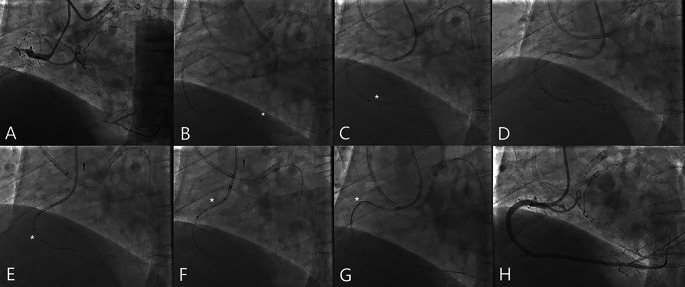
An example of the APT implemented via an epicardial collateral channel in a retro-recanalization CTO case. (**A**) The bilateral angiogram showed a RCA CTO lesion (J-CTO score = 5). (**B**) The RW successfully entered the Guidezilla™ Guide Extension Catheter. The white star showed distal marker of the RM. (**C**) The RM (white star) failed to be pushed into the Guidezilla™. (**D**) Antegrade plaque modification with a 2.0 mm balloon. (**E, F**) The RW and the Guidezilla™ was pulled as the RM (white star) crossed the lesion and entered the Guidezilla™ (black arrow showed the motion of antegrade catheter). (**G**) An antegrade guidewire was successfully inserted into the RM (white star) inside the Guidezilla™. (**H**) The final angiogram showed successful recanalization of the RCA CTO. RW refers to the retrograde wire. RM refers to the retrograde microcatheter. APT refers to the active pulling technique

APT can be performed after the RW crossing the CTO and entering the antegrade guide catheter (Fig. 1A) via the following steps: (1) Anchoring the RW tightly by a suitable balloon or a snare in antegrade guide catheter (Fig. 1B); (2) Disengaging the retrograde guide catheter from the coronary ostium and pulled back 3–4 cm into the aorta to avoid ostial dissection because of retrograde guide catheter retraction during the pulling process (Fig. 1C); (3) Installing a torque device at the entry port of RM to firmly interlock the RW and the RM (Fig. 1F); (4) Pulling antegrade guide catheter outwards with the goal of pulling the RM along with it across the CTO lesion (Fig. 1D), most of the time, in order to protect the CTO vessel from being cut by the RW, an antegrade extension catheter can be positioned in the target vessel, and then the pulling maneuvers can be performed; (5) Re-engaging the antegrade guide catheter and pulling it repeatedly until the RM crossed the occluded lesion and entered the antegrade catheter (Fig. 1E). The schematic diagram was shown in Fig. 1.

### Definitions

Coronary CTO was defined as total occlusion with an occlusion duration of ≥ 3 months [12]. J-CTO score, which consists of entry shape, calcification, bending, occlusion length, and retry lesion, was calculated according to the bilateral angiogram [13]. PCI-related myocardial infarction (MI) was defined according to the fourth universal definition of myocardial infarction (2018) [14] Device success was defined as the RM successfully crossed the CTO lesion in the procedure. Duration of device success was calculated from the RW being advanced and anchored in the antegrade guide catheter to the successful passage of the RM through the CTO lesion. Technical success was defined as a visual assessment of recovery antegrade TIMI flow grade III in the target vessel and residual stenosis < 30%. Process success was defined as technical success without in-hospital major adverse cardiac events (MACEs). In-hospital MACEs included cardiac death, PCI-related MI, ischemia-driven revascularization PCI, or emergency cardiac surgery. Procedural complications included donor vessel injury, target vessel perforation, ostial injury of the target vessel, collateral perforation, and cardiac tamponade.

### Statistical analysis

Continuous variables with normal distribution were presented as mean ± standard deviation and were compared by Student’s *t* test. Categorical variables were presented as frequencies (%). Discrete variables were expressed as percentages (%). The Pearson *χ*^2^ test or Fisher exact test was used to estimate the differences in categorical variables. A *P* value < 0.05 was chosen to indicate a significant difference. All statistical analyses were performed with SPSS 21.0 software.

## Results

### Clinical characteristic

A total of 80 CTO cases who underwent retrograde CTO PCI with the RW successfully crossed the lesion were analyzed. 40 cases who applied the APT were enrolled in the APT group. Another 40 cases were randomly selected from previous CTO-PCI cases before the APT was invented, they were in the non-APT group. The baseline clinical data and the angiographic information of the two groups were similar (Table 1). Table 2 demonstrates the procedural details in both groups. All procedures were finished with 6 or 7 F guide catheters, and the 7 F guide catheter were used more than 50% in the retrograde manner. Since femoral access provides greater guide catheter support, the femoral access accounted for 70.0% and 62.5% of retrograde procedures performed in both groups. The usage rate of antegrade guide extender is significantly higher in the APT group (80% vs. 50%, *P* = 0.005), while the non-APT group employs the retrograde anchor balloon technique more frequently than the APT group (47.5% vs. 25%, *P* = 0.036). The device success rate was higher in the APT group than in the non-APT group ( 100% vs. 85%, *P* = 0.013), with shorter duration (5.3 ± 3.8 vs. 18.6 ± 5.9 min, *P* < 0.001) and less consumption of RMs in the APT group ( *P* < 0.001). The average air kerma radiation exposure was significantly lower in the APT group (2.7 ± 1.2 vs. 4.3 ± 1.7 Gy, *P* < 0.001) than the non-APT group, with shorter fluoroscopy time (69.0 ± 15.0 vs. 88.1 ± 18.9 min, *P* < 0.001) and procedure time (116.2 ± 22.2 vs. 131.6 ± 28.7 min, *P* = 0.009). The technical success rates were 100.0% in both groups. No procedural complications and in-hospital MACEs were adjudicated to the APT group. The process success rate and procedural success rate in the non-APT group were 92.5% and 85%, respectively, the latter being significantly lower than that of the APT group (85% vs. 100%, *P* = 0.013, show in Table 3).

### Two representative cases of APT practice using different types of retrograde collateral

An example of the APT case was illustrated in Fig. 2. A 52-year-old man was transferred to our hospital with exertional angina despite optimal medication treatment. The angiogram revealed a RCA CTO lesion which was failed to be recanalized 3 months ago. With a radial 6 Fr SAL 1.0 guiding catheter (Medtronic) in RCA and a femoral 6 Fr EBU 3.5 guiding catheter (Medtronic) in LCA, the primary retrograde approach was started since the last antegrade attempt resulted in significant diffuse dissection in mid RCA and the collateral channels were prominent (Fig. 2A). After a Sionblack wire (Asahi intecc) and 150 cm microcatheter (APT Medical Inc.) negotiating the septal channel, the reverse CART technique was employed. The retrograde Pilot 150 wire (Abbott) was successfully manipulated into antegrade guiding catheter (Fig. 2B) and was trapped by an antegrade balloon, but the RM failed to cross the CTO body. After fixing tightly between the RW and the RM with a torque device, the antegrade catheter as well as the trapped RW were together pulled outside to provide the RM with antegrade traction to cross the occlusion (Fig. 2C-E). The final angiogram showed successful recanalization of the RCA CTO (Fig. 2F).

**Table 1:** Baseline clinical data and angiographic information of the study population

	APT (*n* = 40)	Non - APT (*n* = 40)	*p*
Age, yrs	59.3 ± 10.8	60.4 ± 9.7	0.922
Gender, male	23 (57.5)	25 (62.5)	0.648
Hypertension (%)	27 (67.5)	29 (72.5)	0.626
Diabetes (%)	13 (32.5)	15 (37.5)	0.639
Hyperlipemia (%)	10 (25.0)	14 (35.0)	0.329
Smoker (%)	19 (47.5)	21 (52.5)	0.655
Prior MI (%)	9 (22.5)	11 (27.5)	0.606
Prior PCI (%)	15 (37.5)	17 (42.5)	0.648
LVEF, %	51.6 ± 10.4	50.8 ± 12.2	0.874
CTO vessel (%)			0.281
LM	0 (0.0)	1 (7.5)	-
LAD	10 (25.0)	12 (30.0)	-
LCX	3 (7.5)	7 (17.5)	-
RCA	27 (67.5)	20 (50.0)	-
CTO assessment (%)			
Length > 20 mm	13 (32.5)	20 (50.0)	0.112
Bending > 45°	28 (70.0)	22 (55.0)	0.166
Calcification	30 (75.0)	12 (60.0)	0.181
Blunt stump	18 (45.0)	22 (55.0)	0.371
Re-try lesion	17 (42.5)	24 (60.0)	0.117
Diffuse lesion in distal vessel	20 (50.0)	25 (65.0)	0.260
Side branch at landing zone	13 (32.5)	19 (47.5)	0.171
Ipsilateral collateral	2 (5.0)	5 (12.5)	0.235
J-CTO score	3.1 ± 1.2	3.0 ± 1.0	0.395

CABG: coronary artery bypass graft; LVEF: left ventricular ejection fraction; MI: myocardial infarction; PCI: percutaneous coronary intervention; CTO: chronic total occlusion; LAD: left anterior descending artery; LCX: left circumflex artery; LM: left main artery; RCA: right coronary artery

**Table 2:** Procedural characteristics of the study population

	APT (*n* = 40)	Non - APT (*n* = 40)	*P*
Antegrade guide catheter size (%)			0.117
6 Fr	17 (42.5)	24 (60.0)	-
7 Fr	23 (57.5)	16 (40.0)	-
Retrograde guide catheter size (%)			0.651
6 Fr	16 (40.0)	18 (45.0)	-
7 Fr	24 (60.0)	22 (55.0)	-
Antegrade guide extender (%)	32 (80.0)	20 (50.0)	0.005
Retrograde guide extender (%)	22 (55.0)	30 (75.0)	0.061
Retrograde anchor balloon technique (%)	10 (25.0)	19 (47.5)	0.036
Access (%)			0.478
Retrograde from radial	12 (30.0)	15 (37.5)	-
Retrograde from femoral	28 (70.0)	25 (62.5)	-
Primary retrograde approach (%)	17 (42.5)	20 (50.0)	0.501
Retrograde conduits used (%)			0.108
Septal channel	28 (70.0)	21 (52.5)	-
Epicardial channel	12 (30.0)	19 (47.5)	-
Retrograde wire technique (%)			0.371
Retrograde wire crossing	4 (10.0)	3 (7.5)	-
Kissing wire technique	11 (27.5)	17 (42.5)	-
Reverse CART	25 (62.5)	20 (50.0)	-
Device success (%)	40 (100.0)	34 (85.0)	0.013
Duration of device success, min	5.3 ± 3.8	18.3 ± 5.9^*^	< 0.001
Number of retrograde microcatheter usage (%)			< 0.001
1	36 (90.0)	4 (10.0)	
2	4 (10.0)	31 (77.5)	
3	0 (0.00)	5 (12.5)	
Types of retrograde microcatheter (%)			0.461
Finecross MG (Terumo)	11 (25.0)	26 (32.1)	
Corsair (Asahi)	19 (43.2)	37 (45.7)	
Instantpass (APT Medical Inc.)	14 (31.8)	18 (22.2)	
Air kerma radiation exposure, Gy	2.7 ± 1.2	4.3 ± 1.7	< 0.001
Fluoroscopy time, min	69.0 ± 15.0	88.1 ± 18.9	< 0.001
Procedure time, min	116.2 ± 22.2	131.6 ± 28.7	0.009
Contrast volume, ml	149.5 ± 36.7	159.0 ± 38.8	0.264

CTO: chronic total occlusion; CART: controlled antegrade and retrograde tracking

^*^ 34 cases achieved device success in this group

**Table 3:** In-hospital MACEs and procedural complications of the patients

	APT (*n* = 40)	Non - APT (*n* = 40)	*p*
Technical success (%)	40 (100.0)	40 (100.0)	1.000
Procedural complications (%)			
Donor vessel injury	0 (0.0)	1 (2.5)	-
Target vessel perforation	0 (0.0)	0 (0.0)	-
Ostial injury of the target vessel	0 (0.0)	0 (0.0)	-
Collateral perforation	0 (0.0)	2 (5.0)	1.000
Cardiac tamponade	0 (0.0)	0 (0.0)	-
Process success (%)	40 (100.0)	37 (92.5)	0.120
In-hospital MACEs (%)			
Cardiac death	0 (0.0)	0 (0.0)	-
PCI-related MI	0 (0.0)	2 (5.0)	1.000
Ischaemia-driven PCI	0 (0.0)	1 (2.5)	-
Emergency cardiac surgery	0 (0.0)	0 (0.0)	-
Procedural success (%)	40 (100.0)	34 (85.0)	0.013

MACE: major adverse cardiac event; MI: myocardial infarction; PCI: percutaneous coronary intervention

Another example of APT manipulation was demonstrated in Fig. 3. A 65-year-old man with severe angina was transferred to our hospital. The patient has a history of chronic kidney failure receiving hemodialysis for 10 years. About five months ago, he underwent a coronary angiogram, and a stent was implanted in LAD. However, the RCA CTO was failed to be recanalized (Fig. 3A). Given the difficulty of antegrade wire crossing (J-CTO score = 5), the retrograde approach was selected as the starting strategy. A radial 7 Fr EBU 3.75 guiding catheter (Medtronic) and a femoral 6 Fr SAL 1.0 guiding catheter (Medtronic) were engaged to the left and right coronary ostia, retrospectively. A promising epicardial collateral channel from diagonal to posterior descending artery (PDA) was used. After successful RW tracing the collateral channel, the reverse CART technique was attempted with an antegrade 2.0 mm × 20 mm balloon (Medtronic), the Guidezilla™ Guide Extension Catheter was advanced to the mid RCA segment to facilitate the RW externalization (Fig. 3B). A retrograde Pilot 200 guide wire (Abbott) was then manipulated and finally entered the Guidezilla™. Then the RW was tightly trapped by the antegrade 2.0 mm × 20 mm balloon (Medtronic) at the Guidezilla™, but it was impossible to push the RM (white star) across the long-segment occlusion (Fig. 3C), even after antegrade plaque modification with the 2.0 mm × 20 mm balloon (Medtronic) (Fig. 3D). Then the APT was initiated when the RW and the RM were “married” by a torque device at the entry port of the RM, the RM (white star) was gradually pulled through the CTO along with the RW (Fig. 3E-F). An antegrade Runthrough NS wire (Terumo) was advanced into the RM (white star, Fig. 3G) in the Guidezilla™, followed by 4 stents implanting from PDA to ostium RCA, and an excellent final angiogram demonstrated the successful reopening of the CTO lesion (Fig. 3H).

## Discussion

### Efficacy of the APT

In the present study, the technical success rate were 100% in both groups, much higher than previous studies [15–17], this may be due to only cases with successful RW crossing the CTO lesion were included. Following the use of APT, all cases in the APT group successfully navigated the RMs through the CTO lesion, with shorter passage time and a smaller number of RMs used, irrespective of the type of RM employed. The outperformance of APT was perhaps because that the “pull” from the antegrade side provides more direct traction to advance the retrograde equipment than a “push” from the retrograde side would. In the frequently-used “trapping balloon technique”, the propulsion of the RM is largely dependent on the support provided by the retrograde system, and this propulsion diminishes progressively as it navigates through the long tortuous collateral, thus making it very difficult for the RM to pass through the occlusion. However, employing APT allows for a proactive application of dragging force directly onto the RM, facilitating its successful passage through the CTO lesion.

While the successful passage of a RM through a CTO lesion is not a mandatory requirement for the success of retrograde CTO PCI, it significantly influences the progression of the procedure. Therefore, the present study revealed that the radiation exposure was lower, the fluoroscopy time and the procedure time were shorter in the APT group than its counterparts. In addition, we observed that it can be highly efficient to switch to the APT immediately after the “trapping balloon technique” failed. For some severe resistant lesions, a more proactive strategy was to perform the APT and the “trapping balloon technique” synchronously with extreme caution, which would greatly improve the efficiency and thus reducing more procedure time. If initial APT is unsuccessful, it can be assisted by antegrade plaque modification and then redo the APT until the RM was pulled through the lesion. In procedures conducted prior to the invention of APT, specifically in the non-APT group of 40 cases, if the “trapping balloon technique” proved ineffective, the most commonly employed alternative approach was to replace the RM with a new one and attempt further advancement. Consequently, this led to an increase in the usage of microcatheters. If this approach was unsuccessful, other methods were considered to complete the procedure, such as Tip-In technique, Intracoronary Rendezvous technique or antegrade wire crossing. However, since the implementation of APT, it can help the RM quickly cross the occlusion without other equipment and extra expenses, thus simplifying the procedure, improving the efficiency and reducing PCI consumables.

### Safety considerations for APT practice

The disadvantage and potential danger of this technique is the increased amount of force and tension applied to the system in three key places: (1) The retrograde guide catheter may be pulled forward aggressively into the ostium of the donor vessel; (2) There is necessarily increased tension in the retrograde collateral system; (3) The RW on the “pull” end of the system (involving the antegrade guide catheter) can be unprotected, which makes the aorta/ostium and proximal segment of this vessel susceptible to a “cheese cutter” injury especially if the pulling guide is not coaxial. In order to reduce potential risks aforementioned, there are several details to pay close attention to during the APT process. First and foremost, before performing APT, make sure retrograde guide catheter had kept a proper distance away from the ostium of the donor vessel to avoid deep throating related ostial injury. Secondly, during the pulling process, the RW should be pulled gradually with continuous fluoroscopy, synchronizing with the heart beat, taking care not to kink it. In the present study, no donor vessel injury and collateral perforation were observed in the APT group, the reason may be that the collateral system is entirely protected by the RM, it is less likely to be injured in the “pulling” procedure. Before the whole procedure ends, the angiogram from the retrograde side should be performed to ensure that there is no injury to the donor vessel and the collateral vessel. Thirdly, to shield the aorta/ostium and proximal segment of target vessel from being “cut”, advancing an antegrade guide extension catheter into target vessel would be a preferable option. We can directly pull the antegrade guide extension catheter instead of pulling the antegrade guide catheter. Besides, the guide extension catheter can provide coaxial alignment, add back-up support, shorten the pulling distance. Therefore, the present study demonstrated a higher utilization rate of antegrade guide extension catheters in the APT group compared to the non-APT group, with the aim of protecting the ostium of the target vessel and avoiding “cutting” injury in the procedure.

### Key notes for APT implementation

First, securing a torque device at the entry port of the RM to “marry” the wire and microcatheter system is of the utmost importance for APT implementation. Without it, this APT idea is only a fantasy. Secondly, violent pulling is strictly prohibited, especially when an aggressive “pull” is applied but no movement made. Under such circumstances, we recommend combining other techniques to facilitate RM crossing the lesion, similar to “push and pull” technique [18] (i.e. pulling the RM from the antegrade side as well as pushing it from the retrograde side), exchange for another RM, plaque modification, support augmentation et al. Thirdly, the APT can be performed in ipsilateral retrograde approach only with the “Ping-pong” guide catheter technique [19]. Last but not least, remember to check the collateral channels and the donor vessel meticulously before the whole procedure ends.

As far as we retrieved, this is an original technique to solve RM-uncrossable lesions in retrograde PCI. In a previous report, Ge et al. described a similar Reverse Wire Trapping technique (RWT) [20]. The RWT differs from the APT because the pulling direction is opposite. The RWT involves a pull from donor vessel side, which would exert a greater force on donor vessel than the APT does, hence the RWT would be more risky than our APT. In some selective CTO patients, the RWT can be a helpful bailout alternative, but it is indeed time-consuming and complicated in practice. In contrast, the APT does not rely on extra devices and is easy to perform, making it a preferable maneuver to these techniques mentioned above.

## Limitations

There remain some limitations of this study. First, this is a single center study with limited number of cases, which may result in sampling bias. Secondly, due to the retrospective design, some causal statistics cannot be compared between the two group. Thirdly, due to the non-random selection of cases in the two groups, and the fact that the enrolled cases are not within the same time period, there may be a potential influence of selection bias.These limitations will highlight the necessity and importance of launching a prospective study with more cases and the randomized controlled design in the future.

## Conclusions

The APT is an easy and safe technique that can greatly improve procedural efficiency without adding other instruments, and allows the RM to quickly crossing the CTO body after successful RW externalization.

## Data Availability

No datasets were generated or analysed during the current study.
