# Correction to: The active pulling technique to solve microcatheter-uncrossable lesions in retrograde chronic total occlusion percutaneous coronary intervention

**DOI:** 10.1007/s10554-024-03224-6

**Published:** 2024-08-19

**Authors:** Hongmin Zhu, Xinyong Cai, Yuliang Zhan, Lang Hong

**Affiliations:** 1 Jiangxi Medical College, Nanchang University, Nanchang, Jiangxi, 330046 China; 2 Department of Cardiology, Jiangxi provincial People’s Hospital, The First Affiliated Hospital of Nanchang Medical College, No. 256, Fenghebei Avenue, Honggutan District, Nanchang, Jiangxi, 330006 China

**Correction to: The International Journal of Cardiovascular Imaging (2024) 40:1019–1027**

10.1007/s10554-024-03068-0

The Funding information section was missing from this article and should have read

Funding Information:

- Funding Name: Interventional Therapy Clinical Medical Research Center of Jiangxi Province
- Grant Number: 20223BCG74005

The original article has been corrected.

